# Practical Notes and Formulæ

**Published:** 1883-02-20

**Authors:** 


### PRACTICAL NOTES AND FORMULÆ.

**New Treatment for Vaginitis.**—M. Terrillon proposes a method of treatment which consists essentially in the introduction into the vagina of the following ointment:

| | | |
|---|---|---|
| ℞ | Ac. tannic | 50 grms, |
| | Amyli | 150 grms, |
| | Ung. petrolei | 150 grms. |

M. This ointment is placed in a sort of speculum, so arranged that the ointment can be forced out as the instrument is withdrawn from the vagina. If the vulva opneing is large a small tampon of cotton may be introduced. Generally from fifteen to twenty grams of the unguent is sufficient at one application, and it need not be repeated for seven or eight days.—*Ex.*

**A Valuable Mixture for Chills.**—A subscriber sends us the following as a valuable mixture for chills, and desires to give it a more extended notice, he being able to heartily recommend it:

| | | |
|---|---|---|
| ℞ | Quinine sulph. } Cinchonidia sulph } | grs. xxx, |
| | Acid sulph | ♏ x, |
| | Liq. potass. arsen | f. ʒ j, |
| | Ext. nucis. vom. fld | ♏ x, |
| | Aquæ q. s. ad | f. ℥ iv. |

M. Tablespoonful every four hours, when the fever is off.—*Pharmacist and Chemist.*

**Bromides and Choral in Whooping Cough.**—M. Dujardin recommends the combination of the bromides and chloral as being very useful in whooping cough. He gives one dessertspoonful of the mixture in a glass of milk, to which the yolk of an egg has been added, evening and morning:

| | | |
|---|---|---|
| ℞ | Potassii bromidi | ʒ ss, |
| | Sodii bromidi | ʒ j, |
| | Ammonii bromidi | ʒ ss, |
| | Ssr. chloral | ℥ ss, |
| | Aquæ | ℥ ij. |

—*Med. Summary.*

**To Correct the Odor of the Lochia After Child-Birth**—

| | | |
|---|---|---|
| ℞ | Acid. carbolici glacial | ℥ j, |
| | Glycerini | ℥ j, |
| | Aquæ puræ | ℥ viij. |

M. Sig. A tablespoonful in eight ounces of warm water twice a day as a vaginal injection.—*Med. Summary.*

**Disguising the Taste of Quinine.**—J. L. Lilly, in the Indiana Pharmacist, says that the following syrup has proved to be very satisfaetory in disguising the taste of quinine:

℞ Fluid extract yerba santa.................... 4 parts,
Water.................................... 8 parts,
Powd. pumice.............................. 1 part,
Granulated sugar.......................... 14 parts.

Mix the fluid extract with the water, evaporate to seven parts, shake with pumice, allow to stand, decant, add sufficient watar to preserve the measure, then with heat dissolve the sugar.—*Med. Summary.*

**Asthma Mixture**—

℞ Tinct. lobeliæ.............................. ℥ v,

Ammonii iodidi............................ ʒ ij,

Ammonii bromidi........................... ʒ iij,

Syr. tolutani.............................. ℥ iij.

M. Sig. Teaspoonful every one, two, three or four hours. This gives relief in a few minutes, and sometimes the relief is permanent.—*Fothergill.*

**Pruritus Vulvæ.**—Dr. Wm. Goodell, the world-famed gynecologist of the university, recommends

℞ Carbolic acid............................... ʒ j,

Morphine sulphate........................... gr. x,

Boracic acid................................ ʒ ij,

Vaseline.................................... ℥ij. M.

for pruritus vulvæ; and, also, the patting of the parts with a sponge soaked in boiling-hot water. This is also a most excellent application for that rawness so often found between the thighs of the newly born.—*E. J. Kempf, M.D., in Med. Herald.*

**Hair Wash.**—The Medical Bulletin says the following will be found very efficacious, slightly stimulating and cleansing to the scalp:

℞ Potassii iodidi ⎫

Pulv. sodii biboratis ⎬ aa ℥ iss

Aquæ ammoniæ ⎪

Tr. lyttæ ves ⎭

Ol. rosmarini............................... ʒ ij,

Ol. myristicæ............................... ℥ ij,

Aq. cologniensis............................ Ojv,

Ol. macidis................................. ℥ iss.

Aquæ........................................ Oviij. M.

—*Medical Age.*

**Tasteless Quinine.**—Dr. Dashiel, in Nashville Medical Journal, says: "In the intermittents of children, I append a formula for a *tasteless* preparation of quinine, which is readily taken by them:

| | | |
|---|---|---|
| "℞ | Quiniæ sulph. | aa ℈ ij, |
| | Pulv. acacia | |
| | Acid tannic | grs. iij, |
| | Syr. simplicis | fl. ℥ ij. |

"M. Sig. Give half to one teaspoonful every two hours, according to age.

"In the chronic intermittents of adults, we usually follow up the arrest of the paroxysms, by the use of one of the following tonics, as they may prefer, a pill or mixture. Their use should be continuously persisted in, at least, for a month:

| | | |
|---|---|---|
| "℞ | Quiniæ sulph | ʒ j, |
| | Sol. arsenit. potass | fl. ʒ j, |
| | Acid sulph. dilut | aa fl. ℥ ij. |
| | Syr. zinziberis | |

"M. Sig. Take teaspoonful three times a day *after* meals. Or the following:

| | | |
|---|---|---|
| "℞ | Acid arseniose | grs. iv, |
| | Strychniæ sulph | grs. ij, |
| | Cinchonidiæ sulph | ʒ iss, |
| | Ferri. sulph. exsic | ʒ iss, |
| | Aloe soc. ext | ℈ j. |

"M. Ft. Pills No. 90. Sig. Take one pill three times a day after meals."

**Treatment of Pertussis.**—M. Dujardin-Beaumetz, in his recently published Lecons de Clinique Therapeutique, recommends the bromides with chloral in the treatment of whooping-cough.

He gives, morning and evening, in a glass of milk containing a yolk of one egg, a dessert or tablespoonful (according to the age of the child) of the following mixture:

| | | |
|---|---|---|
| ℞ | Potass. bromid | ʒ ss, |
| | Sodii bromid | ʒ j, |
| | Ammonii bromid | ʒ ss, |
| | Syrup. chloral. (Fr. cod.) | ℥ iss, |
| | Aquæ | ℥ ij. |

M.—*Med. and Surg. Rep.*

**To Harden the Nipple.**—

| | | |
|---|---|---|
| ℞ | Acid tannic | ℥ iv, |
| | Glycerine | ʒ i, |
| | Aqua ad | ʒ ij. |

This strong solution of tannin acts differently from the weaker combinations of tannin and glycerine, as it actually tans the nipple, making it tough, resolvent and incapable of inflammation.

